# Status of parasitological indicators and morbidity burden of onchocerciasis after years of successive implementation of mass distribution of ivermectin in selected communities of Yeki and Asosa districts, Ethiopia

**DOI:** 10.1186/s12889-020-09344-7

**Published:** 2020-08-12

**Authors:** Gebremedhin Gebrezgabiher, Zeleke Mekonnen, Delenasaw Yewhalaw, Asrat Hailu

**Affiliations:** 1 College of Veterinary Medicine, Samara University, P.O. Box 132, Samara, Ethiopia; 2 School of Medical Laboratory Sciences, Institute of Health Sciences, Jimma University, Jimma, Ethiopia; 3 Tropical and Infectious Diseases Research Center, Jimma University, Jimma, Ethiopia; 4 Department of Microbiology, Immunology, and Parasitology, School of Medicine, College of Health Sciences, Addis Ababa University, Addis Ababa, Ethiopia

**Keywords:** Onchocerciasis, CDTi, Elimination, Skin snip biopsy, Microfilariae, Morbidity, Ethiopia

## Abstract

**Background:**

Control and elimination of onchocerciasis requires regular follow-up and evaluation of community directed treatment with ivermectin (CDTi) program implementation. This research was aimed to assess the epidemiological status of onchocerciasis in disease endemic communities of Asosa and Yeki districts of Ethiopia after 5 and 15 years of successive CDTi respectively, and to evaluate the decline in infection and morbidity burden.

**Methods:**

A community-based cross-sectional study was conducted from August 2017 to January 2018 (i.e. within 2–7 months since the last treatment) using interview, physical and parasitological examinations. Pre-CDTi epidemiological data were obtained from studies conducted prior to the launch of CDTi.

**Results:**

A total of 3002 individuals (1567 from Asosa and 1435 from Yeki) were included. No infection was detected from Yeki. In Asosa, the prevalence of infection was 1.6%. The geometric mean intensity of infection was 0.02 mf/mg of skin snip. The prevalence rates of dermatitis, depigmentation, nodule, and atrophy in Yeki were 33(2.3%), 57(4%), 37(2.6%) and 11(0.7%), respectively. The prevalence rates of papular dermatitis, depigmentation, palpable nodule, atrophy, and blindness in Asosa were 94(6%), 38(2.4%), 30(1.9%), 28(1.8%) and 2(0.1%), respectively. Five years of CDTi had significantly reduced prevalence and intensity of infection by 91.8% (*p* < 0.001) and 99.7% (*p* < 0.001), respectively. Moreover, CDTi reduced prevalence of papular dermatitis by 95.9% (*p* < 0.001), palpable nodule by 90.5% (*p* < 0.001), and atrophy by 30% (*p* = 0.6) in Yeki. Similarly, CDTi reduced prevalence of papular dermatitis by 88.6% (*p* < 0.001), depigmentation by 90.3% (*p* < 0.001), atrophy by 89.5% (*p* < 0.001), and blindness by 90% (*p* < 0.001) in Asosa.

**Conclusions:**

Fifteen years of successive CDTi had brought the infection from high to zero in Yeki. However, thorough entomological and serological data need to be generated to ascertain whether complete interruption of parasite transmission has been attained, and for considerations of an evidence-based CDTi cessation. Five years of CDTi in Asosa has significantly reduced the infection and morbidity of onchocerciasis to very low level. We, hereby, recommend biannual CDTi to continue in Asosa and its surroundings until the infection transmission is fully interrupted.

## Background

Human onchocerciasis (river blindness), caused by the filarial nematode *Onchocerca volvulus*, is a vector-borne parasitic disease of public health and socio-economic concern in the sub-Sahara Africa. It is transmitted by the bite of infected blackfly of the genus *Simulium* which breeds in fast-flowing rivers and streams. The adult worm may live up to 18 years inside nodules located in the body of infected persons [1]. The adult female worm produces thousands of microfilariae (mf) per day [2] that migrate under the dermis of the skin to cause skin and eye diseases. Typical cutaneous lesions of onchocerciasis include acute papular dermatitis, chronic papular dermatitis, lichenified onchodermatitis, lymphadenopathy, depigmentation, and atrophy [3].

Onchocerciasis in Ethiopia was first reported from Bonga by Italian investigators in 1939 [4, 5]. Following this case report, several epidemiological studies have been conducted in Ethiopia, and revealed the presence of the disease in different localities in varying level of endemicity [6–29] According to these studies, the prevalence of the disease ranges from 0% in eastern Ethiopia [28] to as high as 84% in southwest Ethiopia [21]. Consequent rapid epidemiological mappings of onchocerciasis conducted in 1997, 2001, 2004, 2011 and 2012 also proved that the the disease is endemic in several communities in western, southwestern and northwestern Ethiopia [30]. Endemic areas mainly cover the whole Benishangul-Gumuz, portion of Oromia, Amhara, Gambella, and Southern Nations Nationalities Peoples (SNNP) Regions [31]. The clinical picture of the disease is mainly dermal while ocular manifestations are uncommon or absent [4, 6]. It is estimated nearly 5.8 million people are living in highly endemic areas, and over 20 million people are at risk of acquiring infection [32]. The disease has been a major public health problem [21] with socio-economic significance in areas of the country where it is endemic, especially in large-scale coffee plantation enterprises in southwest Ethiopia [11, 16, 17], which are densely populated and vastly covered by forests, with heavy rainfall and numerous perennial rivers and streams [9, 17]. In 2015, onchocerciasis reported to be responsible for 43.9 disability-adjusted life-years lost per 100,000 [33].

As part of the African Program for Onchoceciasis Control (APOC) in sub-Saharan Africa [34, 35], community directed treatment with ivermectin (CDTi) has been the main strategy in the efforts towards the control and elimination of onchocerciasis in Ethiopia [31, 36]. The program was launched in 16 districts of the then Keffa-Sheka Zone of SNNP Region in 2001 [31] and later expanded to other districts where the disease has been reported [32, 36]. Currently, the program is running in 194 endemic districts [37]. As part of the recent renewed interest to eliminate onchocerciasis in Africa, Ethiopia has garnered the concerted efforts of stakeholders in its’ effort to eliminate onchocerciasis.The national elimination program is undertaking 3 major activities: (i) biannual distribution of ivermectin in all CDTi implementation areas, (ii) nationwide mapping of onchocerciasis in untreated areas to detect transmission areas eligible for CDTi and, (iii) post-treatement and post-elimination surveillance activities in CDTi implementation areas that received several rounds of ivermectin treatment. Moreover, the country has also established a committee that provides technical support and decision on the cessation of mass ivermectin treatment, and oversees the overall progress of the elimination program [32, 38, 39].

Control and elimination of onchocerciasis requires regular follow-up and evaluation of program implementation. Then, areas with unsatisfactory progress can be identified quickly [40–42], and corrective actions can be taken. This involves a coverage verification survey, epidemiological, parasitological, serological and entomological evaluations [39]. It is recommended that parasitological evaluation needs are carried out six years after the commencement of ivermectin distribution to observe the decline in infection level in CDTi implementation areas, and if transmission is still ongoing to undertake similar actions after 3 to 4 years till the elimination breakpoint is achieved [34, 42].

**Fig. 1:**
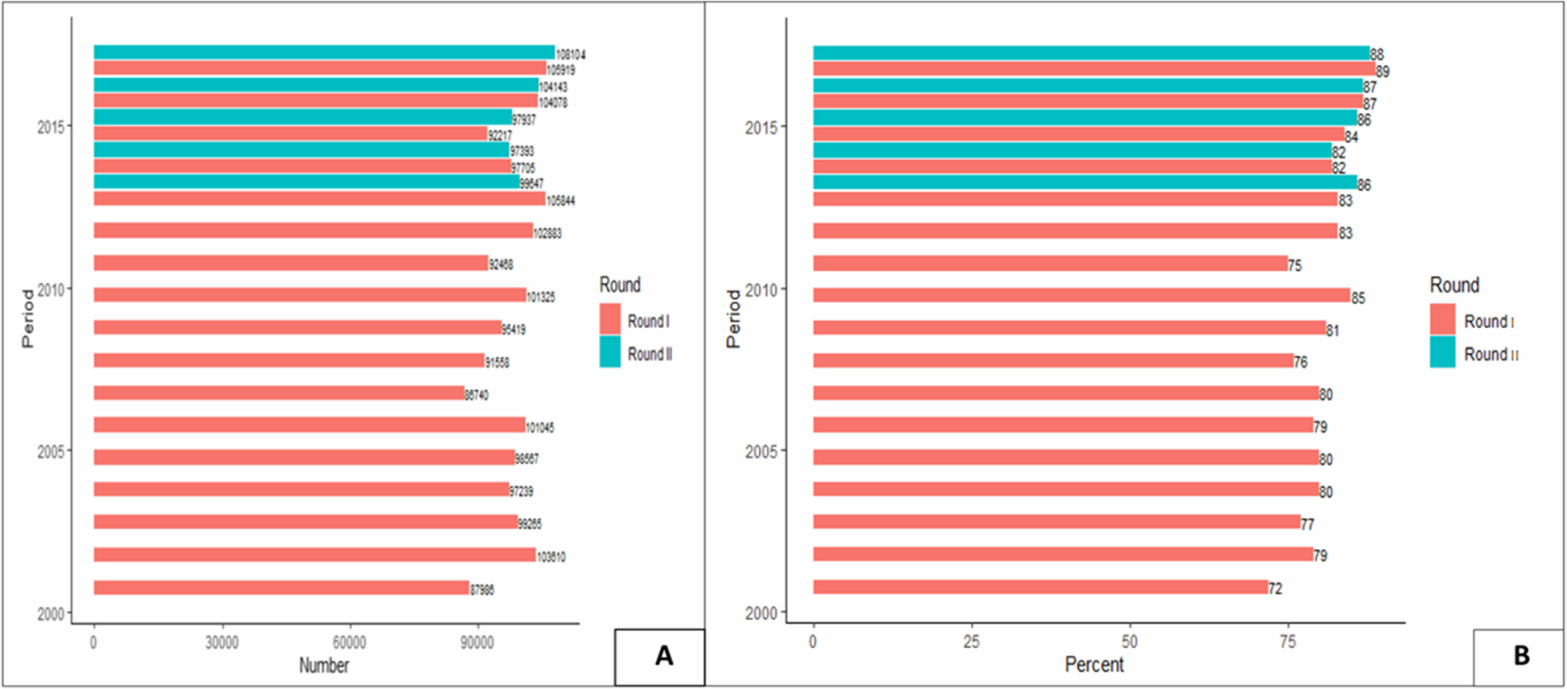
History of ivermectin distribution in Yeki district, Ethiopia (2001–2017). The biannual ivermectin treatment began in 2015, and round I refers to the ivermectin treatment provided in December to January, and round II refers to treatments provided in April to May. **a** Total population treated; **b** Treatment coverage rate among the eligible population

**Fig. 2:**
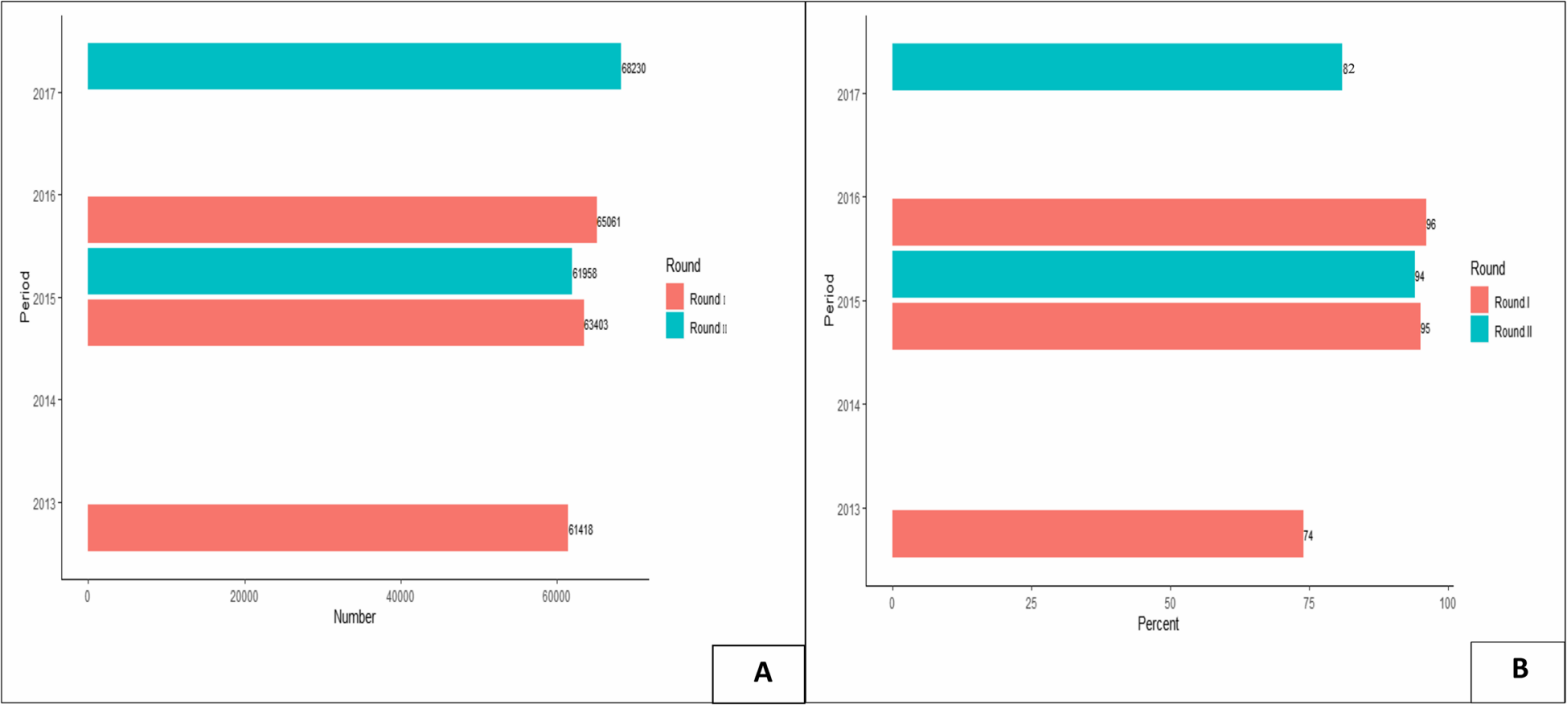
History of ivermectin distribution in Asosa district, Ethiopia (2013–2017). The biannual ivermectin treatment began in 2015, and round I refers to the ivermectin treatment provided in December to January, and round II refers to ivermectin treatment distributed in April to May. **a** Total population treated; **b** Treatment coverage rate among the eligible population. NB: The ivermectin treatment records for the years 2014, 2016 (round II) and 2017 (round I) were not available and documented in the Asosa district health bureau

Pre-control epidemiological studies showed that onchocerciasis was highly endemic in communities of Yeki district [17, 21, 23, 24]. The district is endowed with rapidly flowing perennial rivers, streams, and vegetation cover that provide suitable habitats for the vector. CDTi distribution started in Yeki in 2001 and continued to the present day as part of the national efforts to control the disease in highly endemic areas (Fig. 1). Subsequent parasitological evaluations have shown transmission of the disease was ongoing [34, 43]. Similarly, previous studies indicated that onchocerciasis is endemic in several communities of Asosa [18] and its surroundings [12, 15]. CDTi has been running in this area since 2013 (Fig. 2). However, hitherto, no study was conducted to investigate the impact of the program and to assess the epidemiological status of the disease. The aim of this study was to assess the current status of onchocerciasis in selected communities of Asosa and Yeki districts after 5 and 15 years of successive CDTi program, respectively, and to evaluate the impact of ongoing CDTi activities in reducing the prevalence, intensity of infection and morbidity of onchocerciasis in communities of respective districts that have baseline epidemiological data collected prior to the launch of mass ivermectin distribution.

## Methods

### Study area and design

**Fig. 3:**
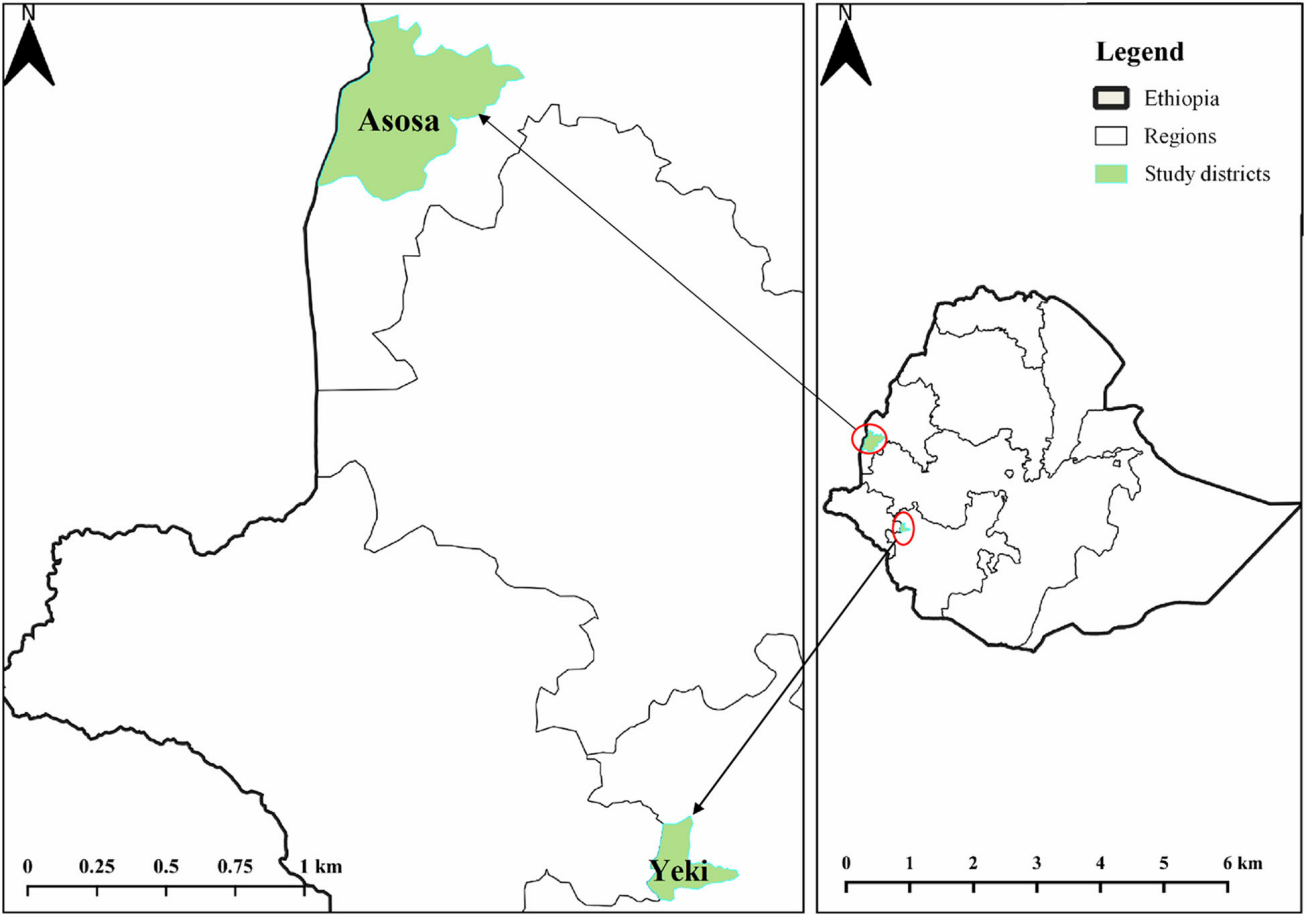
Geographic location of study districts. The study map was produced using QGIS software version 3.10.2 [44]. OpenStreetMap (OSM) shapefiles were downloaded from GADM (https://gadm.org/download_country_v3.html)

The community-based cross-sectional study was conducted from mid August 2017 to the beginning of January 2018 in two onchocerciasis endemic districts namely Yeki of SNNP Region and Asosa of Benishangul-Gumuz Region of Ethiopia (Fig. 3). The study was conducted within 2–7 months since the last mass ivermectin distribution.

Yeki District, located in Sheka Zone of SNNP Region, is bordered by Bench-Maji Zone in the south, Gambella Region in the west, Amderacha district in the north, and Kaffa Zone in the east. There are perennial fast flowing rivers (Beko, Shai, Michi, Opi, Btn, Yambo, Achani, Dingi, Goshenie, Kancha, and Meni) that support the breeding of blackfly. The district is hilly and densely forested, and thus well known for its commercial scale coffee plantation. Yeki district has 22 *kebeles* (the smallest administrative unit) with a total population of 141,539, of whom 70,487 are men and 71,052 are women. The area is inhabited by indigenous people and people who resettled from other highland areas of the country (Wollo, northern Shoa and Tigray) after the two great famines in 1984 and 1985. The dominant ethnic groups residing in this district are Amhara, Shakicho, Kafficho, Sheko, Oromo, Majang, Bench and Manja. Most of the residents are engaged in mixed farming involving coffee plantation, sorghum and maize cultivation, and rearing livestock.

Asosa, one of the districts in Asosa Zone, Benishangul-Gumuz Region, is bordered by Bambasi district in the south, Oda-buldigilu district in the east, Homosha district in the north, Menge district in the northeastern, Kurmuk district in the northwestern, and Republic of Sudan in the west. It is located at an elevation ranging from 500 to 1500 m above sea level. It is 659 km far from Addis Ababa. Fast-flowing rivers that flow in this area include Dabus, Tumed, Andil, Hoha, Salga, Agole, Buldadine and Afa. It is inhabited by indigenous and resettled people. The indigenous people are the Berta ethnic group, residing in small patchily distributed villages. The non-indigenous people are those people that resettled in 1979 in response to the drought that occurred in northern highlands of Ethiopia, mainly Wollo and Tigray. The livelihood of the population is mainly subsistence agriculture and small businesses, e.g. trade. The total population of the district is 109,900. For the entire district, 24,422 households were counted. The district comprises of 74 *kebeles* (Source: Asosa district Health Office).

### Selection of study communities

Communities were purposively selected for this study. Primarily, communities were selected based on the availability of pre-intervention epidemiological data with documented active transmission in Asosa (Megelle 36, Megelle 37, Salga 22, Salga 24, Komeshega 25, Komeshega 26, Hoha 15, Bielmilli, Oorra, and Abramo) [18] and Yeki (Adisberhan and Endris-Gorji) [24]. The infection status from these communities were used to measure the level of decline in infection and morbidity, and to finally estimate the impact of CDTi. Secondly, additional communities located close to fast-flowing rivers and streams were selected for the study. This was done based on (a) Prior observations in communities of the districts while conducting CDTi coverage and compliance rate assessment survey (manuscripts under preparation), (b) Upon a recommendation from health officials and experts of the respective districts. Finally, a total of 38 communities were selected and assessed from the two endemic districts, 15 communities from Yeki and 23 from Asosa.

### Study population

In each study community, all residents aged 15 years and above who willingly came for screening, agreed to give a written consent to undergo physical examination and skin snip procedure were interviewed and examined. The age group with high risk of acquiring infection and most suitable for parasitological evaluation were included for this study [42].

### Data collection

After obtaining the permission to perform the study from community (*kebele*) leaders, selected villages were visited. Community members were mobilized, and gathered at a central point in their village for examination. Then, a detailed discussion was made on the aim of the study, the technique to be used and the importance of being examined for onchocerciasis. Finally, relevant data were collected using the following methods and procedures:

#### Interview

Initially, a questionnaire-based face-to-face interview was made to collect the socio-demographic (personal) information of each study participant including name, sex, age, ethnicity, occupation, and treatment history after obtaining informed written consent (Additional file 1).

#### Morbidity examination

After conducting the interview, physical examination was carried out in a well-lit room to detect the presence of palpable nodules and other signs and symptoms of onchocerciasis.

#### Parasitological examination

Parasitological examinations were performed as previously described [45]. Briefly, following aseptic procedures, skin snips were taken from each side of the gluteal fold in each of the study participants using a disposable pricking needle and sterile razor blade. The skin snips were placed in 96-well microtiter plates containing 100 μl of physiological saline and incubated for 24 h at room temperature. The fluid in the individual well of the microtiter plate was examined, and each mf that has emerged was counted under a microscope [45]. The skin snips from the positive study participants were weighted. Then, the load of infection per mg of skin snip was determined.

### Data analysis

The raw data were first entered into the Microsoft Excel datasheet and validated. Cutaneous signs, nodules and infection prevalences were expressed as the proportion of individuals with morbidities and mf infection respectively. The prevalence and intensity of infection were calculated to evaluate the endemicity level of infection. Prevalence of infection is the percentage of individuals with mf positive skin snip in each district. For positive results, the mf in the two skin snips were counted and the individual mf densities were expressed as the arithmetic mean number of mf/mg of skin snip. For evaluating the intensity of infection geometric mean mf density was used [46]. The microfilarial load for entire population (MFL) and for those aged 20 years and above i.e. community microfilarial load (CMFL) was calculated as in [47]:

$$ MFL\kern0.24em (CMFL)=\mathit{\exp}\left[\sum \limits_1^n\frac{\ln \left( mi+1\right)}{n}\right]-1, $$

Moreover, the arithmetic mean intensity of infection (AMI) was calculated as follows:

$$ AMI=\frac{\sum_1^n mi}{n}, $$

where *m*_*i*_ is the arithmetic mean of the two mf counts per mg of skin snip recorded per study participant *i*, and *n* is the total number of individuals sampled.

A chi-square test was employed to compare the prevalence of infection and the prevalence of morbidity indicators of the disease, and *p*-value less than 0.05 was considered statistically significant.

### Ethical consideration

The study obtained approval from the Institutional Review Board (IRB) of the Institute of Health, Jimma University before the commencement of the study (RPGC/170/06). The aim of the study was explained to the study participants and informed written consent was obtained for those age 18 and above, or by parents or guardians of participants less than 18 years. Permission was obtained from the Regional Health Bureau and/Zonal Health Bureau, District Health Bureau, and *Kebele* Authorities of each study area. Collection of skin snip biopsy was performed with trained professionals. Besides, the study participants were informed that they are free to participate. The collected data were kept confidential in compliance to established Human Subject Protection guidelines.

## Results

### Socio-demographic information of the study participants

In 2017, a total of 141,539 and 109,900 population were reported in Asosa and Yeki districts, respectively. Overall, 3002 individuals were included from the selected communities of both districts. Of these, 1567 (52.2%) and 1435 (47.8%) individuals were from Asosa and Yeki, respectively. The sex ratio was slightly female-biased in Asosa (female proportion of 52.1%), but it was proportional in Yeki (female proportion was 50.4%). The socio-demographic profile of the study participants is depicted in Table 1.

**Table 1:** Socio-demographic profile of study participants from communities of Asosa and Yeki districts, Ethiopia (August 2017–January 2018)

Variable		Asosa		Yeki	
		No examined	No positive (%)	No examined	No positive (%)
Gender	Female	817 (52.1)	8 (1.0)	723 (50.4)	0
	Male	750 (47.9)	17 (2.3)	712 (49.6)	0
Age Group (Years)	15–29	710 (45.3)	1 (0.1)	625 (43.6)	0
	30–44	402 (25.7)	12 (3)	461 (32.1)	0
	45–60	316 (20.2)	7 (2.2)	279 (9.4)	0
	> 60	139 (8.9)	5 (3.6)	70 (4.9)	0
Ethnicity	Amhara	670/(42.8)	0	581 (40.5)	0
	Berta	872 (55.7)	25 (2.9)	–	–
	Oromo	13 (0.8)	0	121 (8.4)	0
	Bench	–	–	79 (5.5)	0
	Kafficho	–	–	232 (16.2)	0
	Manja	–	–	138 (9.6)	0
	Majang	–	–	117 (8.2)	0
	Shakicho	–	–	60 (4.2)	0
	Sheko	–	–	99 (6.8)	0
	Other	12 (0.7)	0	8 (0.6)	0
Occupation	Farmer	576 (36.8)	17 (2.9)	811 (56.5)	0
	Housewife	601 (38.4)	8 (1.3)	401 (27.9)	0
	Student	340 (21.7)	0	198 (13.8)	0
	Other	50 (3.1)	0	25 (1.7)	0

### Prevalence and intensity of infection in communities of Yeki and Asosa

**Fig. 4:**
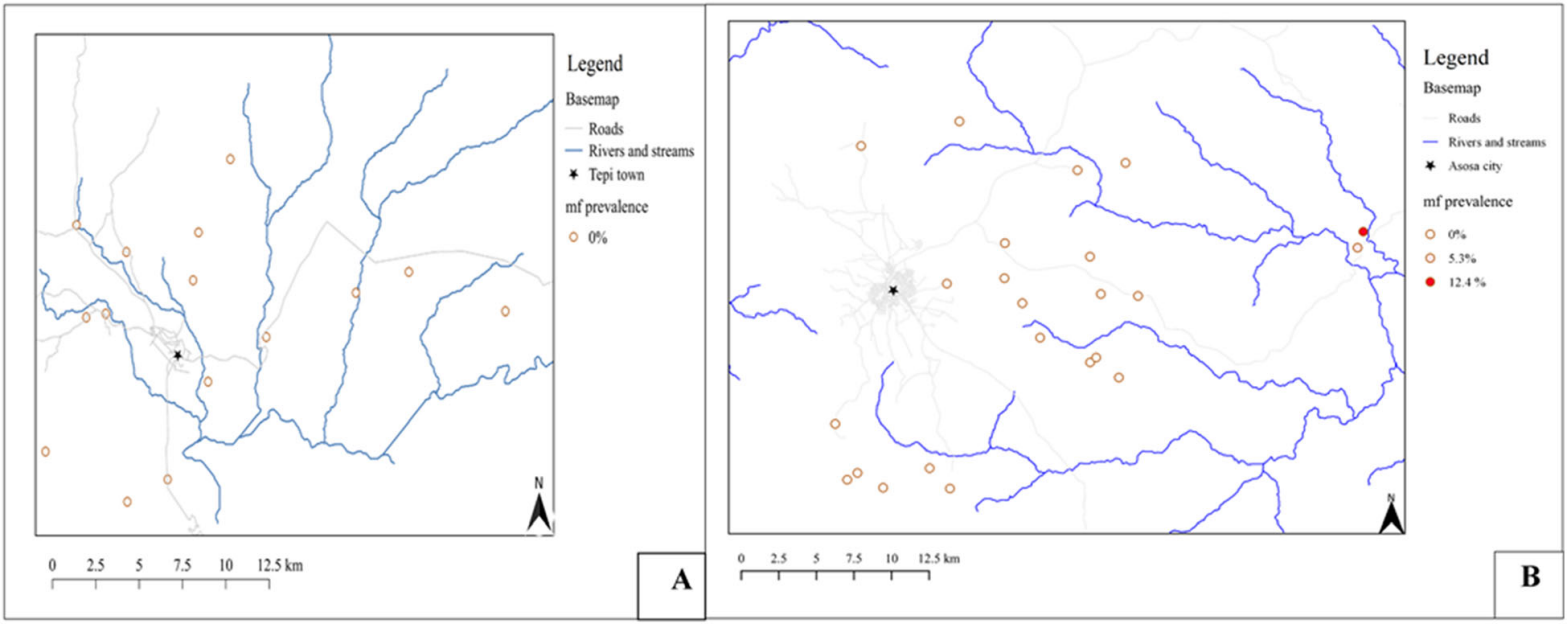
Post-CDTi prevalence of O. volvulus infection in communities of Yeki and Asosa districts, Ethiopia. Maps of point prevalences of onchcocerciasis in the communities of the study districts were produced using the QGIS software version 3.10.2 [44]. OSM shapefilles were obtained from Geofabrik in QGIS software version 3.10.2

No mf was detected in the skin snips of 1435 study participants from Yeki district [95% upper CI: 0.3%]. In Asosa, the overall mf prevalence was 1.6% [95% CI: 1.1–2.3%]; which was 2.3% in male and 1% in female participants. Among the 25 infected, 17 (68.0%) were males. The AMI was 0.05 mf/mg of skin snip. The MFL and CMFL of infection was 0.02 and 0.03 mf/mg of skin snip, respectively (Table 2). The mean weight of the skin snips was 1.9 mg (min: 0.8, max: 2.8, range: 2, and SD: 0.6) among the positives. The prevalence of *O. volvulus* infection in the study communities of the two districts is depicted in Table 2 and Fig. 4.

**Table 2:** Prevalence and intensity of *O. volvulus* infection in communities of Yeki and Asosa districts, Ethiopia (August 2017–January 2018)

District	Community	Population size	No examined	No positive	MFP (%)	AMI	MFL	CMFL	Time since last CDTi (in months)
Yeki	Adisbrehan-EndrisGorji	5412	193	0	0	0	0	0	7.03
	Selamber	7653	90	0	0	0	0	0	6.60
	Shosha	5323	95	0	0	0	0	0	6.96
	Fide	11,020	109	0	0	0	0	0	6.20
	Bechi	13,130	124	0	0	0	0	0	6.03
	Kura	7075	83	0	0	0	0	0	6.83
	Depi	6474	80	0	0	0	0	0	6.30
	Shai	8918	88	0	0	0	0	0	6.80
	Boko	4280	70	0	0	0	0	0	6.73
	Baya-Hbretfrie	8195	104	0	0	0	0	0	6.70
	Darimu	3598	43	0	0	0	0	0	6.57
	Adisalem	5046	93	0	0	0	0	0	6.90
	Zinki	5416	86	0	0	0	0	0	6.53
	Tsanu	4280	47	0	0	0	0	0	6.67
	Kubito	9280	128	0	0	0	0	0	6.30
	Total	105,100	1435	0	0	0	0	0	
Asosa	Hoha 15	974	44	0	0	0	0	0	3.27
	Amba 4	2341	75	0	0	0	0	0	3.57
	Amba 17	1122	52	0	0	0	0	0	3.67
	Megele 38	1469	72	0	0	0	0	0	4.30
	Megele 37	1574	65	0	0	0	0	0	4.37
	Megele 36	818	45	0	0	0	0	0	4.27
	Megele 35	1432	52	0	0	0	0	0	4.27
	Salga 24	1263	53	0	0	0	0	0	4.03
	Salga 22	1151	45	0	0	0	0	0	3.97
	Salga 19	650	52	0	0	0	0	0	4.10
	Komoshega 25	1129	73	0	0	0	0	0	4.17
	Komoshega 26	1317	53	0	0	0	0	0	4.20
	Afasim	1236	82	0	0	0	0	0	3.50
	Oorra	6274	73	0	0	0	0	0	2.47
	Baro	3013	75	0	0	0	0	0	3.87
	Mugufudie	2182	162	20	12.4	0.4	0.2	0.2	4.47
	Dabus-Atinbaqo	974	94	5	5.3	0.2	0.1	0.1	4.43
	Bielmilli	1094	69	0	0	0	0	0	3.80
	Robeyu	1040	52	0	0	0	0	0	3.73
	Tsietsie	2623	73	0	0	0	0	0	2.77
	Aquda	3165	118	0	0	0	0	0	2.97
	Abramo	1940	51	0	0	0	0	0	3.33
	Menge-salga	675	37	0	0	0	0	0	3.13
	Total	39,456	1567	25	1.6%	0.05	0.02	0.03	

*AMI* Arithmetic mean intensity of infection, *CDTi* Community directed treatment with ivermectin, *CMFL* Community microfilarial oad; mf: microfilariae, *MFL* Microfilarial load, *MFP* Microfilarial prevalence

### Prevalence of morbidity indices of onchocerciasis in communities of Yeki and Asosa

**Fig. 5:**
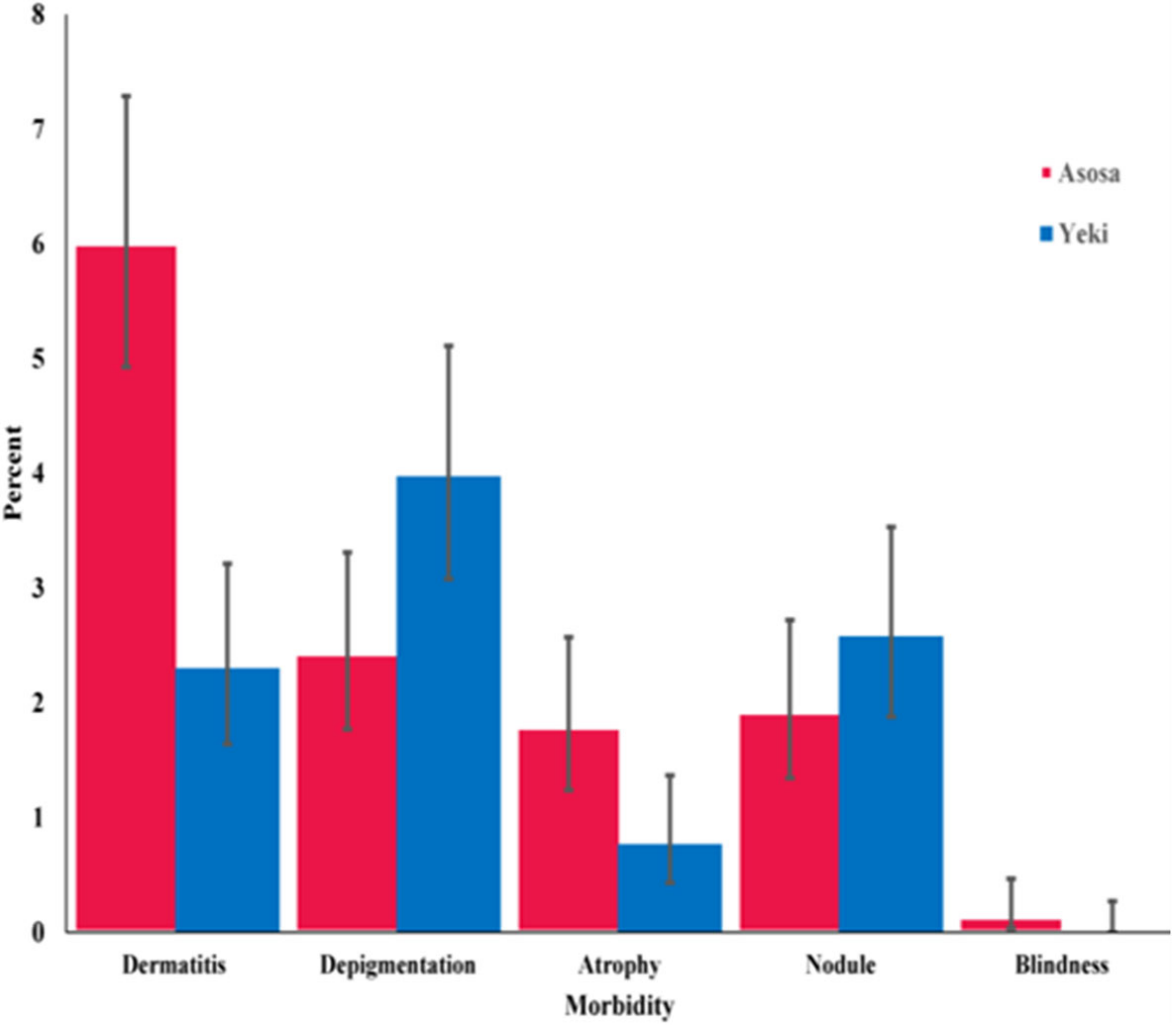
Post-CDTi prevalences of morbidity indicators of onchocerciasis in community of Yeki and Asosa districts, Ethiopia

The prevalence of onchocerciasis morbidity indicators in Yeki were papular dermatitis, 33(2.3%) [95% CI: 1.6–3.2%], depigmentation, 57(4%) [95% CI: 3.1–5.1%], palpable nodule, 37(2.6%) [95% CI: 1.9–3.5%], and skin atrophy, 11(0.7%) [95% CI: 0.4–1.4%]. Similarly, the prevalence of onchocerciasis morbidity in Asosa were, papular dermatitis, 94 (6%) [95% CI: 4.9–7.3%], depigmentation, 38 (2.4%) [95% CI: 1.8–3.3%], palpable nodule, 30 (1.9%) [95% CI: 1.3–2.7%], skin atrophy, 28(1.8%) [95% CI: 1.2–2.6%], and blindness, 2(0.1%) [95% CI: 0.04–0.5%] (Table 3 and Fig. 5).

**Table 3:** Prevalence of morbidities of onchocerciasis in communities of Yeki and Asosa districts, Ethiopia (August 2017–January 2018)

District	Community	Population size	No examined	Morbidity (%)				
				Dermatitis	Nodule	Depigmentation	Atrophy	Blindness
Yeki	Adisberhan-EndrisGorji	5412	193	4 (2.1)	8 (4.2)	14 (7.3)	4 (2.1)	0
	Selamber	7653	90	6 (6.7)	3 (3.3)	5 (5.6)	1 (1.1)	0
	Shosha	5323	95	13 (13.7)	14 (14.7)	8 (8.4)	1 (1.1)	0
	Fide	11,020	109	0	0	1 (0.9)	0	0
	Bechi	13,130	124	3 (2.4)	0	0	0	0
	Kura	7075	83	0	0	0	0	0
	Depi	6474	80	0	1 (1.3)	4 (5.0)	3 (3.8)	0
	Shai	8918	88	0	0	1 (1.1)	0	0
	Boko	4280	70	0	0	1 (1.4)	0	0
	Baya-Hbretfrie	8195	104	4 (3.9)	4 (3.9)	9 (8.7)	2 (1.9)	0
	Darimu	3598	43	0	2 (4.7)	2 (4.7)	0	0
	Adisalem	5046	93	1 (1.1)	1 (1.1)	1 (1.1)	0	0
	Zinki	5416	86	0	1 (1.2)	4 (4.7)	0	0
	Tsanu	4280	47	2 (4.3)	2 (4.3)	3 (6.4)	0	0
	Kubito	9280	128	0	1 (0.8)	4 (3.1)	0	0
	Total	105,100	1435	33 (2.3)	37 (2.6)	57 (3.9)	11 (0.7)	0 (0)
Asosa	Hoha 15	974	44	7 (15.9)	0	0	0	0
	Amba 4	2341	75	0	0	0	0	0
	Amba 17	1122	52	2 (3.9)	0	0	0	0
	Megele 38	1469	72	0	0	0	0	0
	Megele 37	1574	65	0		0	0	0
	Megele 36	818	45	4 (8.9)	0	0	0	0
	Megele 35	1432	52	0	0	0	0	0
	Salga 24	1263	53	3 (5.7)	0	0	0	0
	Salga 22	1151	45	0	0	0	0	0
	Salga 19	650	52	0	0	0	0	0
	Komoshega 25	1129	73	14 (19.2)	0	0	0	0
	Komoshega 26	1317	53	2 (3.8)	0	0	0	0
	Afasim	1236	82	0	0	0	0	0
	Oorra	6274	73	5 (6.9)	0	0	0	0
	Baro	3013	75	11 (14.7)	0	0	0	0
	Mugufudie	2182	162	26 (16.1)	21 (12.9)	28 (17.3)	26 (16.1)	2 (1.2)
	Dabus-Atinbaqo	974	94	7 (7.5)	9 (9.6)	9 (9.6)	2 (2.1)	0
	Bielmilli	1094	69	0	0	0	0	0
	Robeyu	1040	52	0	0	0	0	0
	Tsetse	2623	73	0	0	0	0	0
	Aquda	3165	118	12 (10.2)	0	1 (0.9)	0	0
	Abramo	1940	51	1 (1.9)	0	0	0	0
	Menge-Salga	675	37	0	0	0	0	0
	Total	39,456	1567	94 (6.0)	30 (1.9)	38 (2.4)	28 (1.8)	2 (0.1)

### Comparison of pre and post-CDTi burden of onchocerciasis in Assosa and Yeki

For comparison with the pre-CDTi parasitiological and morbidity burden of onchocerciasis, we also measured the change in the magnitude of intensity, prevalence of infection and morbidity of onchocerciasis. During pre-CDTi survey, 81% mf prevalence was reported in Yeki [24]. In this study, all study participants examined from the same villages had 0% mf prevalence, showing a 100% reduction in pre-CDTi infection. Before CDTi, a mf prevalence of 19.4% and an infection intensity 15.3 mf/mg of skin snip were reported in Asosa [18]. The comparison of infection status of onchocerciasis in the pre-CDTi and post-CDTi period in the study districts is depicted in Table 4.

**Table 4:** Pre and post-CDTi parasitological indicators of onchocerciasis in Asosa and Yeki, Ethiopia

Locality	Parameter	Estimate of parameter		% Reduction	Ref.
		Pre-CDTi	Post-CDTi		
Yeki	MFL	14	0	100	23
	MFP	81	0	100	24
Asosa	MFL	15.3	0.02	99.7	18
	MFP	19.4	1.6	91.8	18

*CDTi* Community directed treatment with ivermectin, *MFL* Microfilarial load, *MFP* Microfilarial prevalence

**Fig. 6:**
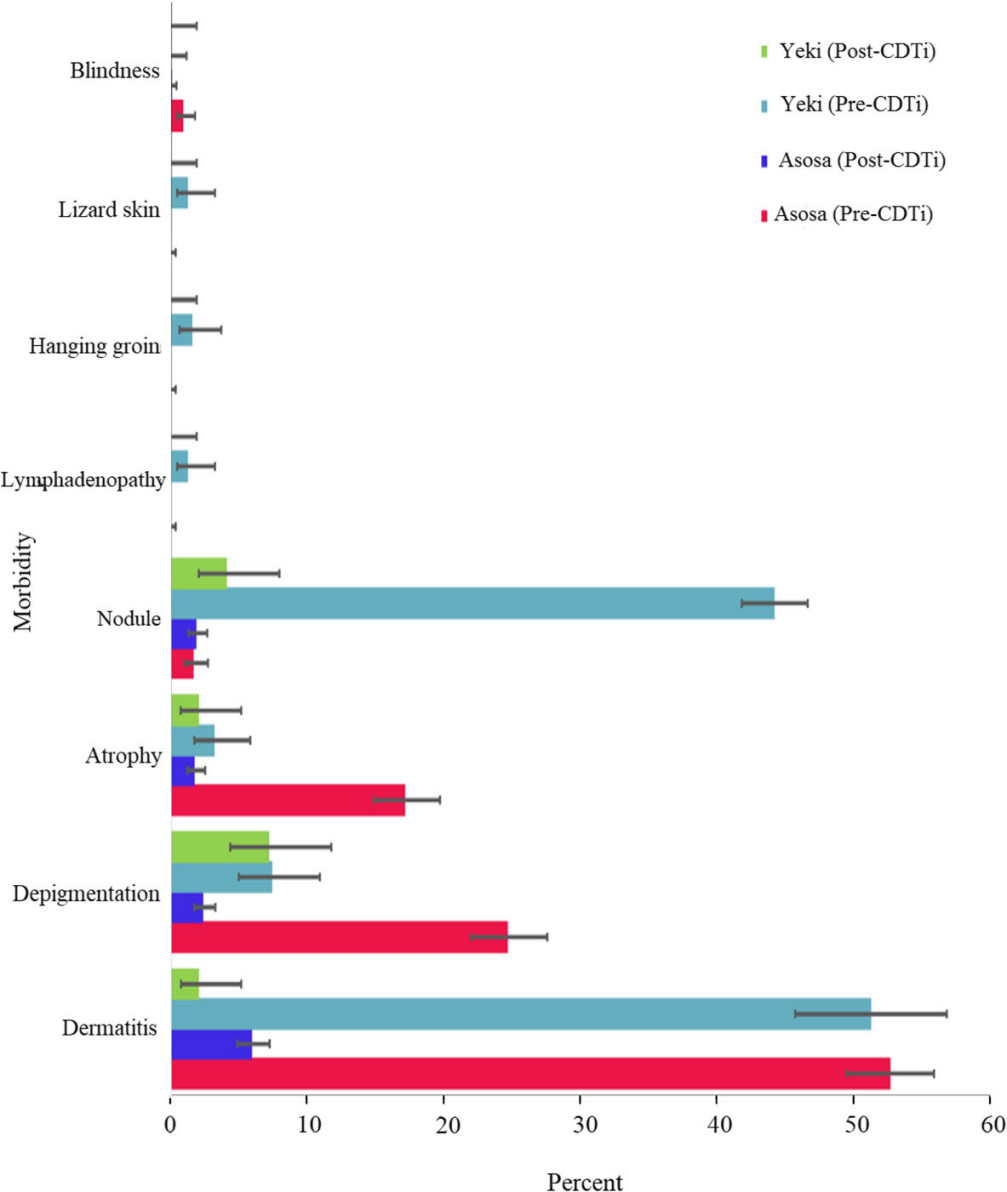
Pre and post-CDTi morbidity burden of onchocerciasis in Asosa and Yeki districts, Ethiopia. It should be noted that the pre and post-CDTi morbidity data presented in Yeki are from Adisberhan-Endris Gorji community only

In the pre-CDTi period, prevalences of palpable nodule (44.2%) [23], papular dermatitis (51%), skin depigmentation (7%), skin atrophy (3%), lizard skin (1%), hanging groin (2%), and lymphadenopathy (1%) were reported in Yeki (Adisberhan-EndrisGorji community) [24]. Following the 15 years CDTi, only 2.1% papular dermatitis, 4.2% palpable nodule, 7.3% depigmentation, and 2.1% atrophy were observed. The CDTi had reduced prevalence of papular dermatitis by 95.9% (*p* < 0.001), nodule by 90.5% (*p* < 0.001) and skin atrophy by 30% (*p* = 0.6). The pre-CDTi prevalence of papular dermatitis, depigmentation, atrophy, palpable nodule, and blindness were 52.7, 24.7, 17.2, 1.7 and 1%, respectively in Asosa [18]. However, the prevalence of papular dermatitis, palpable nodule, depigmentation, atrophy and blindness in post-CDTi was 6, 1.9, 2.4, 1.8 and 0.1%, respectively. The CDTi reduced papular dermatitis by 88.6% (*p* < 0.001); depigmentation by 90.3% (*p* < 0.001), atrophy 89.5% (*p* < 0.001), and blindness by 90% (*p* < 0.001). The magnitude of change in morbidity burden of onchocerciasis in the two districts is depicted in Fig. 6.

## Discussion

This study encompasses the evaluation of phase one onchocerciasis elimination program using a skin-snip microscopy and morbidity assessment survey. We assessed the prevalence, intensity of infection and morbidity of onchocerciasis in selected communities of Yeki and Asosa districts in Ethiopia after 15 and 5 years of successive ivermectin distribution, respectively. We also measured the decline in infection and morbidity of onchocerciasis in communities against pre-control epidemiological data collected before initiation of ivermectin distribution. We also compared our findings with results of previous impact evaluation studies of comparably similar duration of intervention from elsewhere in Africa and Latin America endemic areas.

Before the launch of the CDTi intervention, onchocerciasis was highly endemic in Yeki district. It was a serious public health and socioeconomic problem in this area, particularly in those working in coffee plantation enterprises [17]. The highest pre-control mean mf intensity of infection was reported from this area [17, 23]. This study showed that 15 years of continuous CDTi has brought the prevalence of infection to zero. The skin mf reservoir available to the local vector population in the area might have gradually decreased after the long term treatment [48], and ultimately with possible interruption of active parasite transmission [49]. We believe that the switch from annual to biannual CDTi in 2015 might have contributed to bringing the prevalence and intensity of infection status to zero level. Similar findings were reported from other endemic areas where at least 14 rounds of MDA was carried out in Africa [45, 49–54] and Latin America [55]. In contrast, irrespective of long term CDTi, considerably higher prevalences than the present finding were reported from other CDTi implementation areas in Africa [29, 56–62]. Several factors may have contributed to the differences in the impact of CDTi. Among these include variations in program implementation in the areas (variation in treatment coverage [34, 58, 63, 64]; treatment non-compliance rate [48, 61, 63, 65]; differences in vector species [50, 64], densities [55, 56, 60, 62, 63, 65], biting rates [64], vectorial capacities [64], vector competences [50, 64, 66, 67], vector migration [68–71]; ecological conditions [58, 59, 64, 72]; transmission pattern [50]; parasite susceptibility to ivermectin [73, 74]; human migration [64, 65], and difference in time after community-wide treatment and parasitological assessment). Another possible explanation is that variation in pre-control endemicity level could influence the long term impact of ivermectin on prevalence of infection [34, 50, 51, 57, 64, 75, 76]. In low pre-control endemicity areas, a break in *O. volvulus* infection transmission may be achieved within 6–8 years of treatment [42, 77]. A very good example of this is in Rio Géba hypoendemic focus of Guinea-Bissau where onchocerciasis had been eliminated only after seven years of biannual CDTi [63]. While, in high pre-control endemicity areas, many years of ivermectin treatment may be required to achieve disease elimination [34]. Katabarwa et al. [57] for example refers to Toubouro holoendemic focus of north Cameroon where the highest pre-control endemicity level of onchocerciasis was ever recorded [66], and even 21 years annual CDTi with good coverage had not interrupted transmission of infection [34].

Furthermore, our study demonstrated an overall prevalence and intensity of infection of 1.6% and 0.02 mf/mg skin snip, respectively in Asosa. Interestingly, no mf was detected in the study participants from 21 of the 23 communities (including from all of the study participants of evaluation communities where the disease was reported in the pre-control period). The current prevalence in Asosa was recorded only from two communities; Mugufudie and Dabus-Atinbaqo. This could be due to the presence of a nearby fast-flowing rivers, Dabus, Tumed, Tasa, Gemedie and Andil rivers which serve as conducive sites for blackfly breeding. The residents of Dabus-Atinbaqo and Mugufudie communities predominantly rely on these rivers for their livelihood; and practice irrigation, fishing, crop farming and gold mining on day-to-day basis so that they are frequently exposed to blackfly bites. The study participants from both communities complained that blackfly nuisance and biting is a problem in the environs of their village. The biting rate is reported by residents to be intense especially near to the Dabus River. Relatively higher prevalence of infection than this was reported in other studies after similar years of intervention [43, 49, 65–67, 78–83].

Our study also investigated the magnitude of onchocerciasis associated morbidities in both study areas. Unlike other previous study [78], the observed prevalences of onchocerciasis morbidity were low. Among the observed prevalences, a relatively higher prevalence of papular dermatitis was observed in Asosa. Nonetheless, all the dermatitis might not be caused by onchocerciasis. It could be by other etiologic agents like dermatophytes, contact allergens and insect bites [79, 80]. The findings of this study has revealed the decline to low levels in the magnitude of the morbidities following the CDTi intervention in both study areas. This suggests that repeated and prolonged CDTi could have an impact on the morbidity of onchocerciasis through reducing the progress of existing lesions [81] and preventing the occurrence of new lesions [79].

## Limitations of the study

The findings of this study should be interpreted with the below-mentioned caveats in mind. First, the parasitological evaluation to detect active *O. volvulus* infection is not sensitive enough to detect low levels of *O. volvulus* infection [42, 82, 83] following multiple rounds of treatment [63]. It is also an invasive procedure and there is high refusal in communities participating in the study especially those systematic non-compliers might not be represented in the study [51]. Thus, the findings could be biased [50] and might not exactly signify the exact levels of infection in the communities of the two districts. Second, pre-control epidemiological data were not available for most of the communities in Yeki and Asosa so that it was not possible to observe and compare the impact of mass ivermectin intervention on the epidemiology of the disease at community level. Third, our study began few i.e. two months after the second biannual mass ivermectin distribution. The time gap between treatment and mf assessment has been short (especially in communities of Asosa where the survey started). This may affect the estimates of infection and inturn partly impact informativeness of the study in the progress to disease elimination. Last but not least, there was a long time gap in between the collection of baseline pre-control data and the start of the initiation of ivermectin distribution in Asosa. Thus, it could be possible that the epidemiology of the disease might have been partly changed in the area in the earlier years due to causes other than ivermectin intervention. For instance; this could be supplemented by vectors related factors due to environmental modifications caused by land-use and land-cover changes which inturn might have affected the vector breeding sites. This phenomenon was seen in the Humera area of northwestern Ethiopia where presence of onchocerciasis was reported in 1981 [10], yet repeated evaluation survey in 2009 confirmed the absence of infection without ivermectin intervention [26]. The study participants in Asosa also complained that much of the forest and vegetation has been cleared for crop farming during the resettlement and villagization program.

## Conclusions

Fifteen years of successive CDTi intervention transformed the epidemiological situation of onchocerciasis in Yeki. It brought the mf infection from highly endemic to zero. The fact that no single skin snip positive person was detected suggests that interruption of infection might have been achieved, and the CDTi program is well underway to meet the goal of interrupting parasite transmission in the area. This observation might give clues on the possibilities that interruption of transmission might also have been achieved in other onchocerciasis endemic areas of Ethiopia currently under CDTi intervention. However, thorough entomological and serological evaluations are recommended to ensure whether complete interruption of parasite transmission has been achieved, and for an evidence-based CDTi cessation. Moreover, 5 years of CDTi in Asosa significantly reduced the prevalence, intensity of infection and morbidity of onchocerciasis to very low level.

It is tempting to declare that onchocerciasis is no longer a public health problem in Yeki, and possibly in Asosa. From the elimination point of view, however, there is still ongoing transmission of infection in two communities. We, hereby, recommend biannual CDTi need to continue in Asosa and its surroundings until the transmission of infection is fully interrupted.

## Supplementary information

**Additional file 1.** Interview Guide. This face-to-face interview guide was used to obtain the personal information (socio-demographic profile) of the study participants.

## Data Availability

All data generated during this study are included in this manuscript.

## Abbreviations

AMI: Arthimetic mean intensity of infection
APOC: African Program for Onchocerciasis Control
CDTi: Community Directed Treatment with ivermectin
CMFL: Community Microfilarial Load
mf: Microfilariae
MFL: Microfilarial Load
MFP: Microfilarial Prevalence
OSM: OpenStreetMap
SNNP: Southern Nations Nationalities Peoples
