# Supplementary material for: Status of parasitological indicators and morbidity burden of onchocerciasis after years of successive implementation of mass distribution of ivermectin in selected communities of Yeki and Asosa districts, Ethiopia
Source: BMC Public Health. 2020 Aug 12;20:1233. doi: 10.1186/s12889-020-09344-7 (PMC7425055; doi:10.1186/s12889-020-09344-7)
Supplement: Supplementary file 1 — Additional file 1. Interview Guide. This face-to-face interview guide was used to obtain the personal information (socio-demographic profile) of the study participants. [file 12889_2020_9344_MOESM1_ESM.docx]

## File name: Additional file 1

## Title of data: Interview Guide

## Description of data: This face-to-face interview guide was used to obtain the personal information (socio-demographic profile) of the study participants.

1. District: ___________ *Kebele*: ______________ Village (community): _____________
2. Geographic Coordinates: Altitude: __________ Longitude __________ Latitude ________
3. Sociodemographic information:
   - Name of study participant: _________________________ ID No: _____________
   - Sex: [Female / Male]
   - Age (in years):___________
   - Ethnicity: ___________
   - Religion: _____________
   - Main occupation: __________
   - Length of stay in the community (in years): ___________
   - Number of treatment rounds received: _____________________
